# The status of carbon neutrality of the world's top 5 CO_2_ emitters as seen by carbon satellites

**DOI:** 10.1016/j.fmre.2022.02.001

**Published:** 2022-02-15

**Authors:** Fei Jiang, Wei He, Weimin Ju, Hengmao Wang, Mousong Wu, Jun Wang, Shuzhuang Feng, Lingyu Zhang, Jing M. Chen

**Affiliations:** a Jiangsu Provincial Key Laboratory of Geographic Information Science and Technology, International Institute for Earth System Science, Nanjing University, Nanjing 210023, China; b Jiangsu Center for Collaborative Innovation in Geographical Information Resource Development and Application, Nanjing 210023, China; c Department of Geography, University of Toronto, Toronto, Ontario M5S3G3, Canada; d Frontiers Science Center for Critical Earth Material Cycling, Nanjing University, Nanjing 210023, China

**Keywords:** Net carbon flux, GOSAT, OCO-2, XCO_2_, Atmospheric inversion

## Abstract

China, the Unite States (US), the European Union (EU), India, and Russia are the world's top 5 fossil fuel and cement CO_2_ (FFC) emitting countries or regions (CRs). It is very important to understand their status of carbon neutrality, and to monitor their future changes of net carbon fluxes (NCFs). In this study, we implemented a well-established global carbon assimilation system (GCAS, Version 2) to infer global surface carbon fluxes from May 2009 to December 2019 using both GOSAT and OCO-2 XCO_2_ retrievals. The reductions of flux uncertainty and XCO_2_ bias, and the evaluation of posterior flux show that GCAS has comparable and good performance in the 5 CRs. The results suggest that Russia has achieved carbon neutrality, but the other 4 are still far from being carbon neutral, especially China. The mean annual NCFs in China, the US, the EU, India, and Russia are 2.33 ± 0.29, 0.82 ± 0.20, 0.42 ± 0.16, 0.50 ± 0.12, and -0.33 ± 0.23 PgC yr^−1^, respectively. From 2010 to 2019, the NCFs showed an increasing trend in the US and India, a slight downward trend after 2013 in China, and were stable in the EU. The changes of land sinks in China and the US might be the main reason for their trends. India's trend was mainly due to the increase of FFC emission. The relative contributions of NCFs to the global land net carbon emission of China and the EU have decreased, while those of the US and India have increased, implying the US and India must take more active measures to control carbon emissions or increase their sinks. This study indicates that satellite XCO_2_ could be successfully used to monitor the changes of regional NCFs, which is of great significance for major countries to achieve greenhouse gas control goals.

## Graphical abstract

**Figure:**
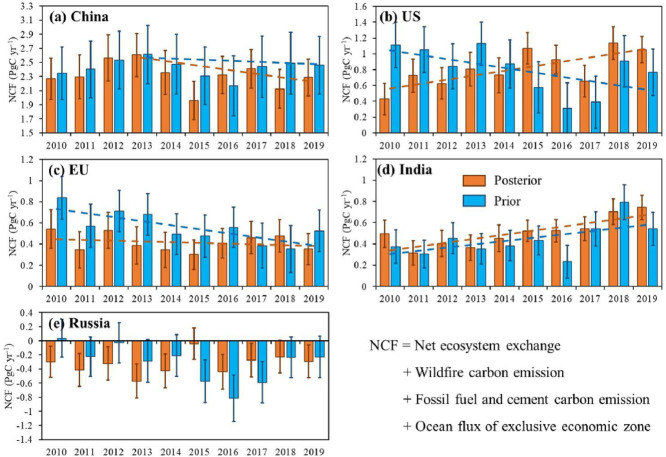

## 1 Introduction

The increase of atmospheric carbon dioxide (CO_2_) concentration, caused by emissions from fossil fuel burning and land use change, is the main cause of global warming [1]. In response to global warming, 175 countries around the world signed the Paris Agreement in 2016, promising to reduce CO_2_ emissions and control global warming within 1.5 °C. Terrestrial ecosystems could uptake CO_2_ from the atmosphere through photosynthesis, and offset the anthropogenic CO_2_ emissions. In the past half century, about 60% of the anthropogenic CO_2_ emissions were absorbed by the terrestrial ecosystems and oceans [1]. In recent years, the European Union (EU), the United States (US), Japan, and Brazil have successively pledged to achieve the goal of carbon neutrality by 2050, and the Chinese government promised to adopt effective policies and measures to curb carbon emissions and to achieve carbon neutrality by 2060. Most recently, the India government also promised to achieve carbon neutrality by 2070 at the United Nations Conference on Climate Change 2021 (COP26). The carbon neutrality means reaching a balance between emitting carbon to the atmosphere (source) and absorbing carbon from the atmosphere (sink), i.e. the net carbon flux (NCF) of a region being equal to zero. The NCF is the sum of fossil fuel and cement (FFC) and wildfire carbon emissions, biosphere and coastal ocean (exclusive economic zone) carbon fluxes located in one region. China, the US, the EU, India, and Russia are the world's top 5 FFC emitting countries or regions (CRs), thus facing huge pressure to reduce their FFC emissions [2].

In the past three decades, a series of studies about the NCFs in Russia, the US, China, South Asia (SA), and Europe were conducted with bottom-up and/or top-down approaches [3], [4], [5], [6], [7], [8]. Bottom-up approach estimates terrestrial net biosphere exchange (NBE) based on forest inventory, grassland resource, and agricultural statistics, or using terrestrial biosphere models (TBMs) [9]. The Strategic Priority Project of Carbon Budget of the Chinese Academy of Sciences systematically investigated the carbon storage and distribution of China's terrestrial ecosystems (forests, grasslands, shrubs, and farmlands). More than 17,000 samples and 200,000 data were collected. The average annual carbon sequestration of the terrestrial ecosystems was about -0.2 PgC yr^−1^ from 2001 to 2010, which was equivalent to offsetting 14.1% of China's FFC emissions during the same period [9]. The Second State of the Carbon Cycle Report (SOCCR2) [8] showed that the US had an annual terrestrial carbon sink of -0.36 PgC yr^−1^ from 2004 to 2013, which offset 24% of its FCC emissions. Top-down approach estimates surface CO_2_ fluxes using globally or regionally distributed atmospheric CO_2_ concentration observations and atmospheric chemistry transport models [10]. Based on surface CO_2_ measurements, Jiang et al. [11] estimated that China's NBE could offset 19% FFC emission from 2001 to 2008 using a nested inversion system, while Wang et al. [12] also estimated a very large carbon sink in biosphere, which offset 45% of the FFC emission during 2010–2015. Combining both bottom-up and top-down estimates, Piao et al. [5] pointed out that China's terrestrial ecosystems absorbed 28–37% of its cumulated FFC emission during the 1980 and 1990 s. With the use of the corrected atmosphere- and land-based estimates as a dual constraint, Janssens et al. [4] estimated a net carbon sink between -0.135 and -0.205 PgC yr^−1^ in Europe's terrestrial biosphere, offsetting 7 to 12% of the 1995 anthropogenic carbon emission.

Top-down approaches have robust performance in carbon budget estimation on global or hemisphere scales with surface CO_2_ observations [13]. However, at regional scales, due to the uneven distribution of *in situ* CO_2_ observations, the reliability of inversion results varies greatly in different regions [14]. Satellite-based measurements of column-averaged CO_2_ dry air mole fraction (XCO_2_) provide global coverage at high spatial resolutions and have improved the top-down estimates of surface CO_2_ sinks and sources at global and regional scales [15], [16], [17], [18], [19]. For example, Takagi et al. [20] found that satellite XCO_2_ retrievals could significantly reduce the uncertainties of surface carbon fluxes for regions in Africa, South America, and Asia, where the surface observations are sparse. Moreover, studies also showed that satellite XCO_2_ retrievals could help to better capture the inter-annual variabilities of terrestrial carbon flux, and well reveal the impact of extreme droughts and large-scale climate anomalies on regional and continental terrestrial carbon dynamics [19,[21], [22], [23], [24]]. In this study, our aim is to explore the potential of using XCO_2_ retrievals to improve the estimates of surface carbon fluxes in the top 5 FFC emitting CRs, and subsequently to assess the mean NCFs of the 5 CRs during the past decade as well as their trends. We conducted a NCF inversion using the Global Carbon Assimilation System (GCAS, Version 2), which has been successfully implemented with GOSAT XCO_2_ retrievals [19]. Both GOSAT and OCO-2 XCO_2_ retrievals were used in this study. In the top 5 FFC emitting CRs, the performance of GCAS was evaluated against independent surface CO_2_ observations, the uncertainty reductions were analyzed, and the mean NCFs during the past decade and their variation trends were investigated.

## 2 Data and method

### 2.1 The global carbon assimilation system (GCAS)

The version 2 of GCAS was developed at Nanjing University in China [19], which mainly focuses on inferring surface carbon fluxes at grid scale using satellite XCO_2_ retrievals within a Bayesian framework (Eq. 1):

(1) $$J\left(X\right)=\frac{1}{2}{\left(HX-y_{obs}\right)}^{T}R^{-1}\left(HX-y_{obs}\right)+\frac{1}{2}{\left(X-X^{b}\right)}^{T}P^{-1}\left(X-X^{b}\right)$$

where **H** denotes an atmospheric transport model, which is the Model for Ozone and Related Chemical Tracers, version 4 (MOZART-4) [25] in this study; *X* denotes the carbon flux to be estimated, $y_{obs}$ is satellite XCO_2_ observations, and **H***X* represents modeled XCO_2_ at the time and location of each XCO_2_ observation. $X^{b}$ is a prior flux. ***R*** and ***P*** are error covariances of model-data mismatch and prior flux, respectively. The modeled XCO_2_ ($XCO_{2}^{m}$) is a vertically integration of the simulated CO_2_ concentration profile according to Eq. 2:

(2) $$XCO_{2}^{m}=XCO_{2}^{b}+\sum_{j}h_{j}a_{j}\left(A\left(c\right)-c_{b,j}\right)$$

where *j* denotes the retrieval level; *c* is the simulated CO_2_ profile, and *A* is a mapping matrix that interpolates *c* into the satellite retrieval levels; $XCO_{2}^{b}$ is a priori XCO_2_; *h_j_* denotes pressure weighting function, *a_j_* and *c_b, j_* are satellite column averaging kernel and prior CO_2_ profile for retrieval, respectively. Following Jiang et al. [19], the model-data mismatch error ***R*** is constructed using the XCO_2_ retrieval error. All retrieval errors were uniformly inflated by a factor of 1.9, but a lowest error of 1 ppm was adopted in this study. In addition, since the retrieval errors of OCO-2 XCO_2_ over ocean (basically smaller than 0.5 ppm) are much smaller than those over land, and are different from those of GOSAT XCO_2_. The OCO-2 XCO_2_ retrieval errors over ocean were further increased by 2 ppm. $XCO_{2}^{b}$, *h_j_, a_j_, c_b, j_* and the retrieval error are provided along with the XCO_2_ product.

There are 4 types of prior carbon fluxes in the GCAS system, namely terrestrial ecosystem carbon exchange (NEE, $X_{NEE}$), wildfire carbon emission (FIRE, $X_{Fire}$), FFC emission ($X_{FFC}$), and CO_2_ exchanges over the ocean surface (OCN, $X_{OCN}$). In this study, the NEE, FIRE, and FFC are combined as land flux ($X_{land}$, Eq. 3). The $X_{land}$ and $X_{OCN}$ are optimized separately, namely $X^{b}$ includes $X_{land}^{b}$ and $X_{OCN}^{b}$. Briefly, $X_{land}^{b}$ and $X_{OCN}^{b}$ from different regions of the globe are inferred within their respective uncertainties, by matching the modeled XCO_2_ with satellite XCO_2_ observations, within their uncertainties. The NCF of one CR consists of the $X_{land}^{b}$ located in its terrestrial region and the $X_{OCN}^{b}$ located in its exclusive economic zone (excluding overseas zone):

(3) $$X_{land}=X_{FFC}+X_{FIRE}+X_{NEE}$$

By minimizing the cost function J(X), we can obtain the posterior flux $X^{a}$, as shown in Eqs. 4 and 5:

(4) $$X^{a}=X^{b}+K\left(y_{obs}-HX^{b}\right)$$

(5) $$K=PH^{T}{\left(HPH^{T}+R\right)}^{-1}$$

***K*** reflects relationships between the changes of surface fluxes in each region and the variations of atmospheric CO_2_ concentrations at each location. Eqs. 4. and 5 are solved using the Ensemble square root filter (EnSRF) algorithm [26].

**Fig. 1:**
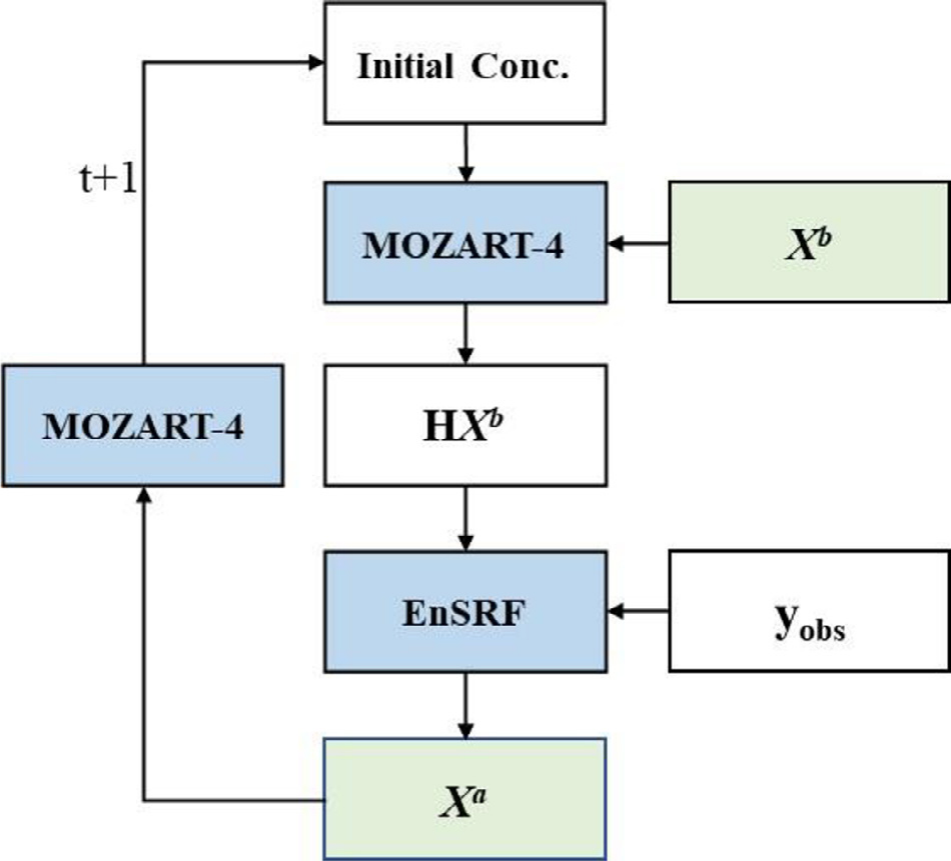
**Flowchart of GCASv2 in each assimilation window**.

In GCAS, the length of assimilation window is one week. The flowchart of each window is shown in Fig. 1. In each DA window, a “two‐step” scheme is employed, in which the prior fluxes $X^{b}$ are optimized using XCO_2_ data in the first step, and whereafter, the optimized fluxes $X^{a}$ are put again into the MOZART-4 model to generate the initial fields of the next DA window. In the first step, according to Eq. 6, $X^{b}$ is perturbed to generate an ensemble (size *n*, here 50) of $X_{i}^{b}$ with a Gaussian random distribution $\delta_{i}$ and a set of scaling factors λ. $\delta_{i}$ has a mean of 0 and a standard deviation of 1, and λ represents the uncertainty of each prior flux. Here, the λ of $X_{land}$ and $X_{OCN}$ were set to be 6 and 10, respectively, which are corresponding to a global 1-δ uncertainty of 1.0 and 0.2 PgC yr^−1^ for the net land flux and the ocean flux, respectively:

(6) $$X_{i}^{b}=X^{b}+\lambda \;\times \delta_{i}\times X^{b},\;i=1,\;2,\;\ldots ,\;n$$

(7) $$P=\frac{1}{n-1}\sum_{i=1}^{n}\left(X_{i}^{b}-X^{b}\right){\left(X_{i}^{b}-X^{b}\right)}^{T}$$

(8) $$X_{i}^{a}=X^{a}+\left(X_{i}^{b}-X^{b}\right)-\tilde{K}H\left(X_{i}^{b}-X^{b}\right)$$

(9) $$\tilde{K}={\left(1+\sqrt{R\;/\;HPH^{T}+R}\right)}^{-1}K$$

Ensemble runs of MOZART-4 were conducted to simulate CO_2_ concentrations. The error covariance of prior flux ***P*** was calculated based on the perturbed flux of $X_{i}^{b}$ following Eq. 7, and ***K*** was calculated based on the ensemble simulations following Eq. 5. To reduce the computational cost and the influence of representative error of XCO_2_, a ‘super-observation’ approach was also adopted in GCAS based on the optimal estimation theory [27]. A super-observation is generated by averaging all observations located within the same model grid within a DA window. In addition, a localization technique was applied to determine which super-observations will be used for the current grid's optimization. It is based on the correlation coefficient between the simulated concentration ensembles in each observation location and the perturbed fluxes in current model grids, and their distances. For details, please refer to Jiang et al. [19]. In this study, GCAS was run from May 1, 2009 to Dec 31, 2019. Two forward simulations with the prior and posterior fluxes were also conducted with the same period. For both assimilation and forward runs, the initial field of 3-D CO_2_ concentrations at 00:00 UTC May 1, 2009 was obtained from the product of CT2017. The first 8 months were considered as a spin-up run, and the results from Jan 1, 2010 to Dec 31, 2019 were analyzed and evaluated in this study. MOZART-4 was driven by the GEOS-5 meteorological fields, which has a spatial resolution of 1.9° × 2.5°, and vertical level of 72 layers. MOZART-4 was run with the same spatial resolution of GEOS-5, but with 56 vertical levels (i.e., the bottom 56 layers of GEOS-5).

### 2.2 Satellite XCO_2_ retrievals

The Greenhouse Gases Observing Satellite (GOSAT) launched in 2009 [28] was developed jointly by the National Institute for Environmental Studies (NIES), the Japanese Space Agency (JAXA) and the Ministry of the Environment (MOE) of Japan, which was designed to retrieve total column abundances of CO_2_ and CH_4_. The Orbiting Carbon Observatory 2 (OCO-2) was launched in 2014, and it is the National Aeronautics and Space Administration (NASA)’s first mission dedicated to retrieving atmospheric CO_2_ concentration. In this study, the GOSAT XCO_2_ retrieval is the ACOS Version 9.0 Level 2 Lite product [29] at the pixel level during May 2009 - Dec 2019, and the OCO-2 XCO_2_ retrieval is the ACOS Version 10.0 [30,31] from Jan 2015 to Dec 2019, which were both bias-corrected and generated using similar retrieval algorithm [32]. Both GOSAT and OCO-2 data were obtained from the data archive at the NASA Goddard Earth Science Data and Information Services Center [33].

The GOSAT and OCO-2 XCO_2_ retrievals have a resolution of 10.5 and 2.9 km^2^ at nadir, respectively. Considering the facts that individual pixel-level retrievals close in time and space are likely to be strongly correlated, and the resolution of the global transport model we used is significantly lower than the pixel of XCO_2_ retrievals, we re-grided the XCO_2_ data into 1° × 1° grid cells following the approach of Wang et al. [18]. The pixel level XCO_2_ data were filtered with xco2_quality_flag (QF), which is the quality flag denoting the science quality of data (0=Good, 1=Bad), and provided along with the XCO_2_ product. In each 1° × 1° grid, all XCO_2_ data with QF equals 0 were selected and averaged. The other variables in the XCO_2_ product like column-averaging kernel, retrieval error, etc., were also re-grided using the same method.

**Fig. 2:**
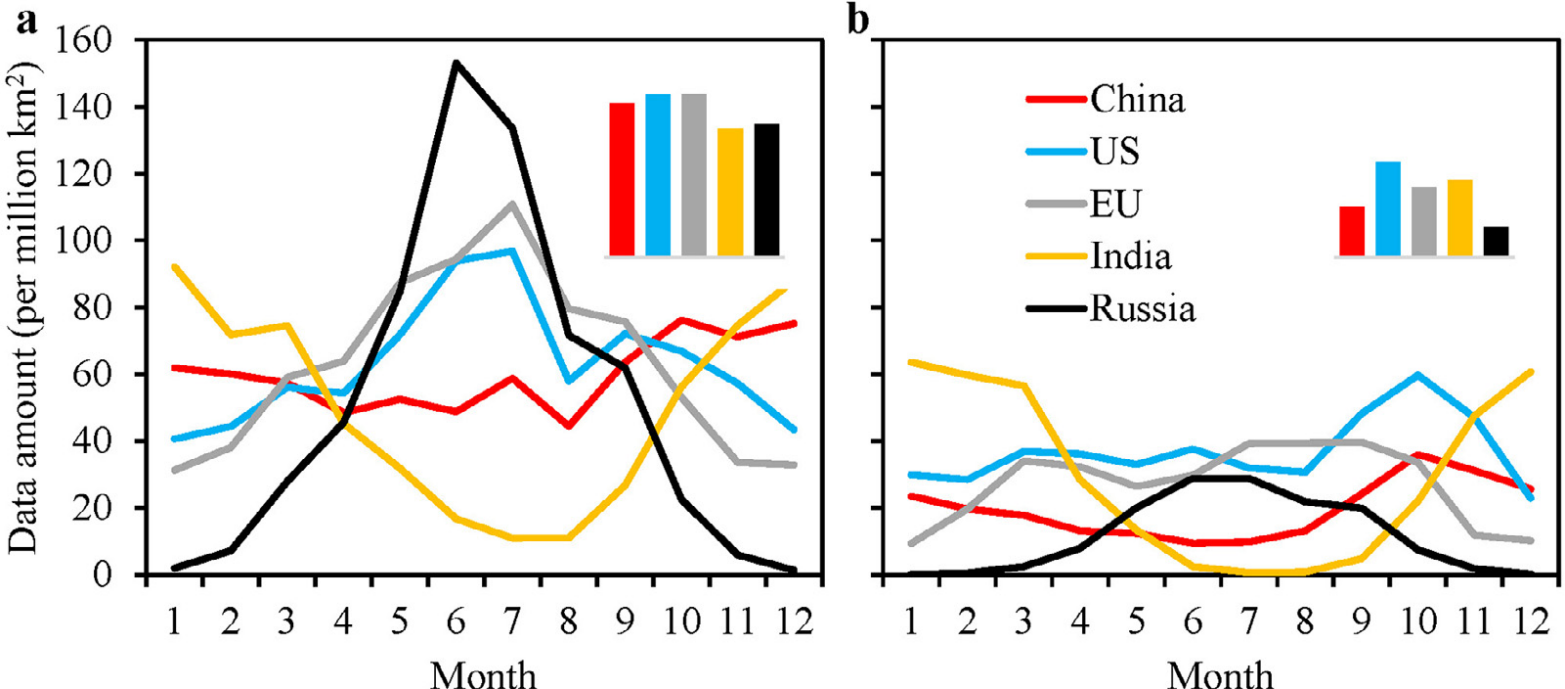
**Monthly variations of data amount per area for (a) OCO-2 XCO_2_ retrievals (averaged from 2015 to 2019), and (b) GOSAT XCO_2_ retrievals (averaged from 2010 to 2019) in the top 5 CRs, the small histogram shows a comparison for the annual data amount among the 5 CRs**.

The amount of data per region is important in the inversion [18]. Fig. 2 shows the monthly data amount per unit area (million km^2^, about 100 of 1° × 1° grid cells) of re-grided XCO_2_ in the 5 CRs during the study period. Overall, after filtering and re-gridding, the data amount of OCO-2 is almost twice that of GOSAT. Although OCO-2 has more XCO_2_ data than GOSAT, our previous study [18] has shown that the carbon flux retrieved based on OCO-2 is not necessarily better than that of GOSAT. Moreover, based on both GOSAT and OCO-2 data, Liu et al. [34] has created a global NBE product, in which the NBE from 2010 to 2014 was inferred from GOSAT, and that from 2015 to 2018 was inferred from OCO-2. The data amounts in Russia and India have significant seasonal amplitudes, while in the other three CRs, the seasonal amplitudes are much weaker. Russia has relatively sufficient data in the warm season and very little data in the cold season, while for India, it is opposite. Generally, the retrieving CO_2_ from space using reflected sunlight is limited by cloud contamination, dark surfaces at shortwave infrared wavelengths, and low solar illumination conditions [29]. The low amount of XCO_2_ in the cold season in Russia is due to the high dark surfaces and low solar illumination conditions, while the low amount of data in India from June to September is due to the frequent cloud coverage in the rainy season. This indicates that during the winter in Russia and the rainy season in India, the constraints of satellite observations on carbon flux may be insufficient to a certain extent. The annual total amount of OCO-2 data are equivalent in these 5 CRs, while those of GOSAT data are relatively large in the US, India, and the EU. On average, there are 25 grids of GOSAT and 57 grids of OCO-2 data in each 100 grids and each month, which is sufficient for carrying out inversions of carbon fluxes.

### 2.3 Prior carbon fluxes

As described in Section 2.1, the prior carbon fluxes consist of NEE, FIRE emission, FFC emission and OCN flux. NEE was simulated using the Boreal Ecosystems Productivity Simulator (BEPS) model [35,36]. The details about the BEPS simulations please refer to Jiang et al. [19]. FIRE emission was from the simulations of SiBCASA model based on the Global Fire Emissions Database version 4 (GFEDv4) [37], which was taken from CT2019B [38], the latest release of the NOAA's CarbonTracker (CT) system [39]. FFC emission was also obtained from CT2019B, which is an average of two products from Carbon Dioxide Information Analysis Center (CDIAC) [40] and Open-source Data Inventory of Anthropogenic CO_2_ (ODIAC) [41], respectively. OCN flux was from the pCO_2_-Clim prior of CT2019B, which was derived from the Takahashi et al. [42] climatology of seawater pCO_2_. The CT2019B dataset spans from January 2000 to February, which is in a spatial resolution of 1° × 1°, and temporal interval of 3 h. It should be noted that for the OCN flux, there is no data in many offshore areas like Japan Sea, Mediterranean, Gulf of Mexico, and East China Sea [42]. Limited by the method, if a region does not have data in the prior flux, we cannot perturb the carbon flux in that region, and calculate the posterior carbon flux through observational constraints. Following Jiang et al. [19], the fluxes in 2009 modeled using a global ocean circulation and biogeochemistry model was used to fill the areas with missing data. In addition, the CT2019B product is only available until the beginning of 2019. For the prior fluxes in 2019, FIRE emission was directly downloaded from the database of GFEDv4.1 s [37], which has a resolution of 0.25° × 0.25°, and time interval of 3 h. OCN flux of 2019 was assumed to be the same as that of 2018. FFC emission was adjusted from the emission in 2018 by a ratio of 2019/2018 in each country or region, which was calculated based on the National Carbon Emissions 2020v1.0 compiled by the Global Carbon Budget 2020 (GCP2020) [2]. We divided global land into 24 regions, including 3 regions (Canada, US, and the rest) in North America, 3 regions (Northern South America, Brazil, and Southern South America) in South America, 6 regions (North, East, West, South and Southeast Europe, and Russia) in Europe (including the Asian part of Russia), 10 regions (China, Mongolia, South Asia, Indo-China Peninsula, Korean peninsula-Japan, Southeast Asian Islands, etc.) in Asia, 1 region in Oceania, and 1 in Africa. In each region, we assumed the distribution of FFC was the same as that of 2018, and adjusted it using the emission ratio of 2018 to 2019.

### 2.4 Evaluation data

Generally, due to the large difference in the spatial scale between the inverted and directly observed flux, direct validation for the optimized flux is impossible. The posterior fluxes were indirectly evaluated by comparing the forward simulated atmospheric CO_2_ mixing ratios against independent CO_2_ measurements, which were obtained from the obspack_co2_1_GLOBALVIEWplus_v6.0_2020-09-11 product (OBSPACKv6) [43]. It contains a collection of discrete and quasi-continuous measurements at surface, tower, aircraft, and ship sites contributed by national and universities laboratories around the world. In this study, flask CO_2_ measurements from 11 surface sites were selected to evaluate the posterior CO_2_ concentrations, in which 2 sites are located in China, 1 site in Russia, 4 sites in the US, and the remaining 4 sites are located in the EU (Table 1). There is only 1 site in India in the database of OBSPACKv6, but the observations are only until January 2013, therefore, it was not chosen in this study. All the 11 sites were provided by the NOAA Global Monitoring Laboratory (with lab number of 1 in each filename). Besides, the GOSAT XCO_2_ retrievals as described in Section 2.2 were also used to test the performance of the assimilation system in the 5 CRs.

**Table 1:** **Site information in the 5 CRs used in this study**.

Country/Region	Station Code	Lat	Lon	Height (masl)	Start month	End month	Record number
China	lln	23.47° N	120.87° E	2862	201001	201912	395
China	wlg	36.29° N	100.90° E	3810	201001	201912	549
EU	cib	41.81° N	4.93° W	845	201001	201912	431
EU	hun	46.95° N	16.65° E	248	201001	201912	495
EU	oxk	50.03° N	11.80° E	1022	201001	201906	380
EU	pal	67.97° N	24.11° E	565	201001	201912	469
Russia	tik	71.59° N	128.88° E	19	201108	201809	334
US	key	25.66° N	80.15° W	1	201001	201912	428
US	nwr	40.05° N	105.58° W	3523	201001	201912	716
US	sgp	36.61° N	97.49° W	314	201001	201912	1102
US	uta	39.90° N	113.72° W	1327	201001	201912	473

## 3 Results and discussion

### 3.1 Evaluation for the inversion results

#### 3.1.1 Global atmospheric CO_2_ growth rates

**Fig. 3:**
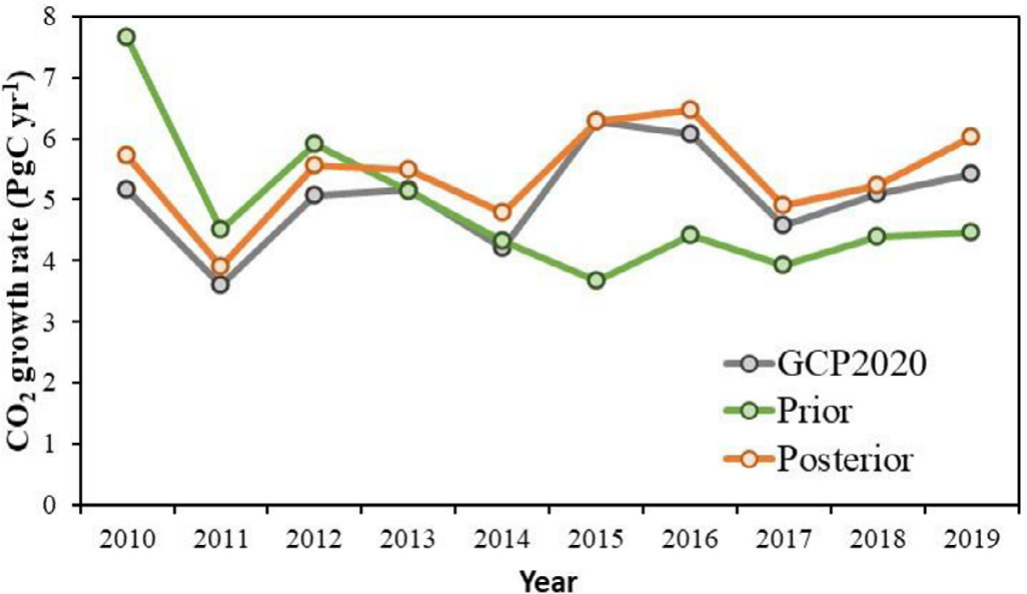
**Interannual variations in the atmospheric CO_2_ growth rates**.

Global atmospheric CO_2_ growth rates (AGRs) could be observed by global background CO_2_ observation sites, which could be used to evaluate the performance of our system on the global scale. Here, we obtained the AGRs from GCP2020, which were provided by the US National Oceanic and Atmospheric Administration Earth System Research Laboratory (NOAA/ESRL) [44]. Fig. 3 shows the interannual variations of the observed, prior, and posterior AGRs during 2010 - 2019. The prior AGRs are significantly higher than the observed before 2013, whereas after 2014, the prior AGRs are markedly lower than the observed. The posterior AGRs have good consistency with the observed values, with small positive biases in each year. The mean observed, prior, and posterior AGRs are 5.06, 4.84, and 5.44 PgC yr^−1^, respectively, and the mean absolute error between the observed and modeled AGR is reduced from a prior value of 1.1 PgC yr^−1^ to a posterior of 0.37 PgC yr^−1^.

#### 3.1.2 Evaluation against atmospheric CO_2_ observations

**Fig. 4:**
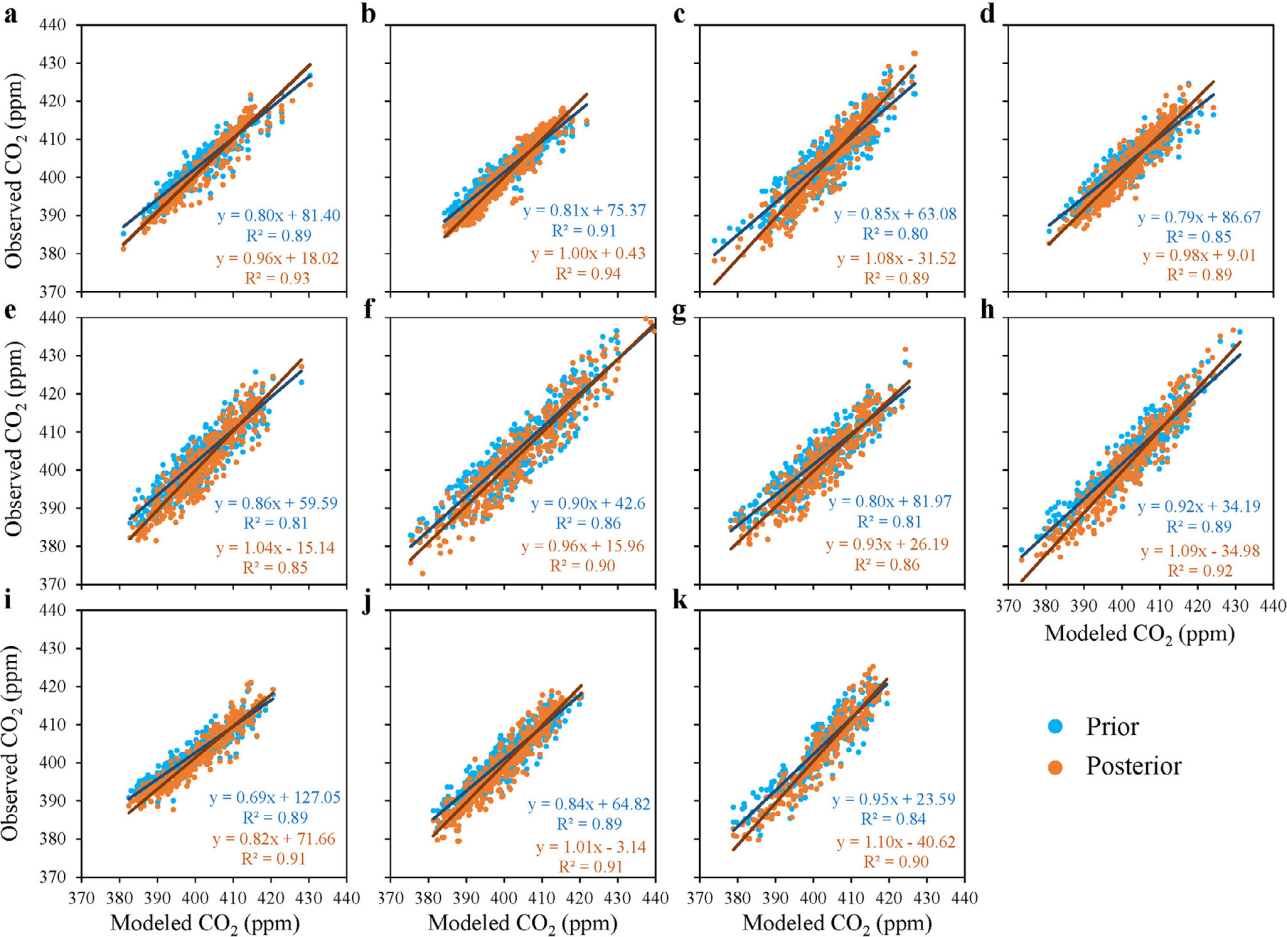
**Scatter plot of the observed and modelled CO_2_ concentrations (a, key; b, nwr; c, sgp; d, uta; e, cib; f, hun; g, oxk; h, pal; I, lln; j, wlg; k, tik)**.

The comparisons of the observed and modelled CO_2_ concentrations at the 11 flask sites are shown in Fig. 4. The modelled CO_2_ driven with prior and posterior carbon fluxes, are respectively named as prior CO_2_ and posterior CO_2_ in the following text. It should be noted that the records with absolute errors between observed and prior CO_2_ concentrations greater than 10 ppm were removed, because they are considered lacking regional representativeness and our model cannot reproduce such observations. Compared with flask observations, the prior CO_2_ concentrations have slopes in the range of 0.69–0.95 among the 11 sites, in which 6 sites are in the range of 0.8-0.9 and only 3 sites are greater than 0.9, suggesting that the prior CO_2_ is basically greater than the observed CO_2_. The posterior CO_2_ has slopes in the range of 0.82–1.1 and less than 0.9 at only 1 site. For the correlation coefficient (*R^2^*), the prior CO_2_ has *R^2^* in the range of 0.80–0.91, and only 1 site has *R^2^* greater than 0.9. The posterior CO_2_ has *R^2^* in the range of 0.85 - 0.94, and there are 7 sites with *R^2^* greater than 0.9. For each site, the slope and *R^2^* of posterior CO_2_ are greater than those of prior CO_2_. These indicate that compared with the prior CO_2_, the consistency between the posterior CO_2_ and the observation is greatly improved. Statistics show that the mean bias (MB) between simulated and observed CO_2_ is reduced from a priori 1.01–2.10 ppm to a posteriori -0.46–1.3 ppm. The Root Mean Square Error (RMSE) of each site is also significantly reduced, with a relative decrease of 8 to 33%. The overall decrease rates of RMSE in China, the US and Russia are comparable. Although the EU has the lowest MB, its mean decrease rate of RMSE is the smallest (Table 2).

**Table 2:** **Statistics of the simulated surface CO_2_ and XCO_2_ concentrations against the surface flask observations and GOSAT retrievals, respectively**.

Country/Region	Site code	MB (ppm)		RMSE (ppm)		*R^2^*	
		Posterior	Prior	Posterior	Prior	Posterior	Prior
China	lln	1.12	2.24	3.01	4.13	0.91	0.89
	wlg	-0.32	1.01	2.55	2.88	0.91	0.89
	XCO_2_	0.00	1.18	0.65	1.91	0.99	0.97
EU	cib	-0.05	1.76	3.56	4.08	0.85	0.81
	hun	0.04	1.67	4.04	4.95	0.90	0.86
	oxk	-0.46	1.07	3.74	4.30	0.86	0.81
	pal	-0.09	1.39	3.33	3.62	0.92	0.89
	XCO_2_	0.67	1.88	0.85	2.34	0.99	0.97
Russia	tik	0.76	2.09	3.41	4.29	0.90	0.84
	XCO_2_	0.21	1.55	0.69	2.23	0.99	0.97
US	key	0.50	1.95	2.21	3.32	0.93	0.89
	mwr	0.04	1.08	1.99	2.69	0.94	0.91
	sgp	0.61	1.18	3.64	4.25	0.89	0.80
	uta	1.30	2.10	3.01	3.73	0.89	0.85
	XCO_2_	0.40	1.47	0.58	1.94	1.00	0.98
India	XCO_2_	0.36	1.38	0.77	1.98	0.99	0.98

Since there are no surface CO_2_ observations in India, we used GOSAT XCO_2_ retrievals to illustrate the performance of our system in these 5 CRs. Monthly regional mean retrieved and modeled XCO_2_ were compared, and the statistical results are shown in Table 2 as well. In order to be more representative, for each CR, we only compared those months with more than 10 re-grided GOSAT XCO_2_ retrievals. It could be found that the RMSE in the 5 CRs of both prior and posterior CO_2_ are comparable, and the RMSE of prior and posterior CO_2_ are about 2 ppm and 0.7 ppm, respectively, with a mean relative decrease of 66%. These indicate that in the GCAS system, the satellite XCO_2_ observations have similar constraints on carbon fluxes in the 5 CRs.

#### 3.1.3 Uncertainty reductions

**Fig. 5:**
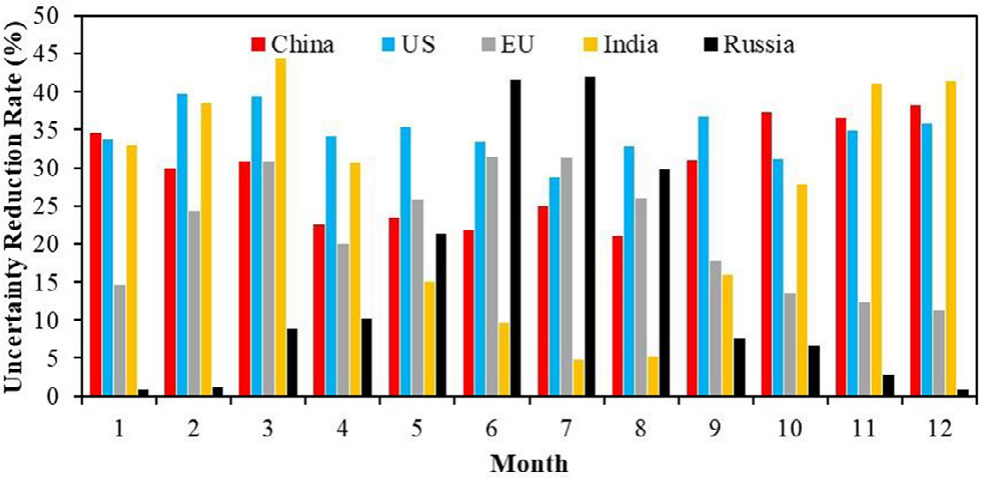
**Monthly uncertainty reduction rate (averaged from 2010 to 2019)**.

The uncertainty reduction rate (URR) is another important metric to evaluate the performance of our system and the effectiveness of the XCO_2_ retrievals [19,45]. Following Chevallier et al. [45], the 1-σ URR is defined as $URR=(1-\sigma_{posterior}/\sigma_{prior})\;\times \;100$, where $\sigma_{posterior}$ and $\sigma_{prior}$ are the posterior and prior uncertainties, respectively, which are standard deviations of the prior and posterior perturbations of $X_{i}^{b}$ and $X_{i}^{a}$ as described in Section 2.1. Due to the uneven distribution of the XCO_2_ data amount, the URRs vary significantly over time and space [19]. Fig. 5 shows the monthly URRs in the 5 CRs averaged during 2010 –2019. Overall, in each CRs, the monthly variations of the URRs are basically consistent with the data amount as shown in Fig. 1, but with lower seasonal amplitude. The US has the weakest seasonal amplitude and highest annual URRs, followed by China and the EU. Both India and Russia have apparent seasonal variations of URRs. India has higher URRs during the cold season and lower ones in the warm season, while Russia has the opposite season variations of URRs. Overall, the annual URRs in China, the US, the EU, India, and Russia are 29.9%, 34.2%, 20.2%, 21.3%, and 25.5%, respectively, indicating that the URRs among the 5 CRs are comparable. The highest monthly URRs of 44.3% occurred in March in India, which is in the range of previous studies (40–70%) [17,19].

### 3.2 Mean NCF during 2010-2019

Table 3 lists the mean prior and posterior NCFs during 2010–2019 in the 5 CRs. The mean prior NCFs in China, the US, the EU, and India are carbon sources, with the strongest in China, followed by the US and the EU, whereas Russia is a carbon sink. Constrained with XCO_2_ retrievals, the sources in China and the EU are reduced, while in India, the source is increased and becomes greater than that in the EU. The sinks in Russia further increases. Little changes occur for the NCF in the US. Except for India, the coastal ocean fluxes act as carbon sinks in the other 4 CRs, but the magnitudes are all very small. These suggest that Russia has achieved carbon neutrality, i.e. its biosphere and coastal ocean carbon sink is greater than its FFC emission, which is not surprising, since Russia has the largest land area and large areas of forest, peat- and wetlands. Dolman et al. [6] estimated the NBE of Russia using inventory-based, eddy covariance and inversion methods, and reported that its NBE was in the range of -0.342 – -1.35 PgC yr^−1^, with the mean of -0.613 PgC yr^−1^, which was also greater than its FFC emission. In addition, Ciais et al. [46] gave a state-of-the-art bottom-up estimate for the NBE of Russia for the period of 2000–2009, and shown that its NBE was -0.725 ± 0.223 PgC yr^−1^. Excluding coastal ocean sink, the lower bound of this estimate (-0.502 PgC yr^−1^) has been greater than its FFC emission (Table 3), also supporting that Russia has achieved carbon neutrality. However, the other 4 CRs are still far from being carbon neutral, especially for China, whose NCF is about 3 times that of the US, more than 4 times those of the EU and India. Taking the available FFC estimates as the truth, we estimate the biosphere and coastal ocean carbon sinks in China, the US, the EU, and India respectively offset 16.7, 42.8, 51.7, and 11.5% of their FFC emissions.

**Table 3:** **Mean prior and posterior NCF during 2010–2019 in the 5 CRs (PgC yr^−1^)**.

Country /Region	Prior flux					Posterior flux			CT2019B⁎⁎	CMS-Flux⁎⁎
	NEE	FIRE	FFC	OCN	NCF	Land*	OCN	NCF		
China	-0.38	0.04	2.79	-0.020 ±0.008	2.45±0.42	2.34±0.29	-0.014 ±0.008	2.33±0.29	2.40	2.47
US	-0.59	0.02	1.44	-0.067 ±0.022	0.87±0.31	0.88±0.20	-0.057 ±0.022	0.82±0.20	1.07	0.96
EU	-0.26	0.00	0.87	-0.061 ±0.017	0.62±0.21	0.48±0.16	-0.060 ±0.017	0.42±0.16	0.90	0.63
India	-0.17	0.04	0.57	0.004 ±0.004	0.44±0.15	0.50±0.12	0.004 ±0.004	0.50±0.12	0.53	0.76
Russia	-0.84	0.13	0.47	-0.071 ±0.017	-0.24±0.29	-0.27±0.23	-0.058 ±0.017	-0.33±0.23	-0.25	-0.52

⁎ The total flux of NEE, FIRE and FFC.

⁎⁎ The fluxes of CT2019B and CMS-Flux exclude ocean flux, which are averaged from 2010 to 2018.

The NCFs over terrestrial region of China, the US, and the EU estimated in this study are lower than the estimates of CT2019B, which was constrained using global surface and aircraft CO_2_ observations, while in India and Russia, our estimates are comparable to those of CT2019B. The largest difference in the estimates between this study and CT2019B is in the EU. CT usually estimated a very weak terrestrial carbon sink (nearly neutral) in Europe, which was significantly weaker than the other estimates using surface observations (-0.2 – -0.44 PgC yr^−1^) [14,[47], [48], [49]] and satellite retrievals (-0.6 – -1.8 PgC yr^−1^) [15], [16], [17]. The NASA Carbon Monitoring System Flux (CMS-Flux) [34] inferred surface CO_2_ fluxes from satellite XCO_2_ retrievals as well, but with FFC prescribed. Although CMS-Flux shows much stronger sources than this study in China, the US, the EU and India, and a much stronger sink in Russia, it also estimated a higher source of NCF in India than that of the EU. Compared with other studies, the SOCCR2 [8] estimated that the US had a mean NCF of 1.14 PgC yr^−1^ during 2004–2013, which is higher than the estimate of this study, probably due to the decrease of FFC (dropped about 0.1 PgC yr^−1^) and the higher estimate of NBE with satellite observations [19]. Based on bottom-up and/or top-down approaches, Patra et al. [50], Thompson et al. [51], and Cervarich et al. [7] estimated the NBE in SA at -0.147 ± 0.239, -0.07, and -0.12 PgCyr^−1^ during 2000s, respectively, with a mean of -0.11 PgCyr^−1^. India accounts for 72 % of the land area of SA. Assuming an even distribution of carbon sinks in SA and the same NBE in the 2010s as in the 2000s, the NCF in India during 2010s was estimated to be 0.49 PgC yr^−1^, which agrees well with our estimate. Excluding some particularly large results [12], China's carbon sink estimated in previous studies was in the range of -0.25 – -0.48 PgC yr^−1^ [5,[51], [52], [53], [54]], with a mean of about -0.37 PgC yr^−1^. Combined with the FFC emission in this study, it can be estimated that China's NCF in the 2010s was 2.42 PgC yr^−1^, which is also close to our estimate.

**Fig. 6:**
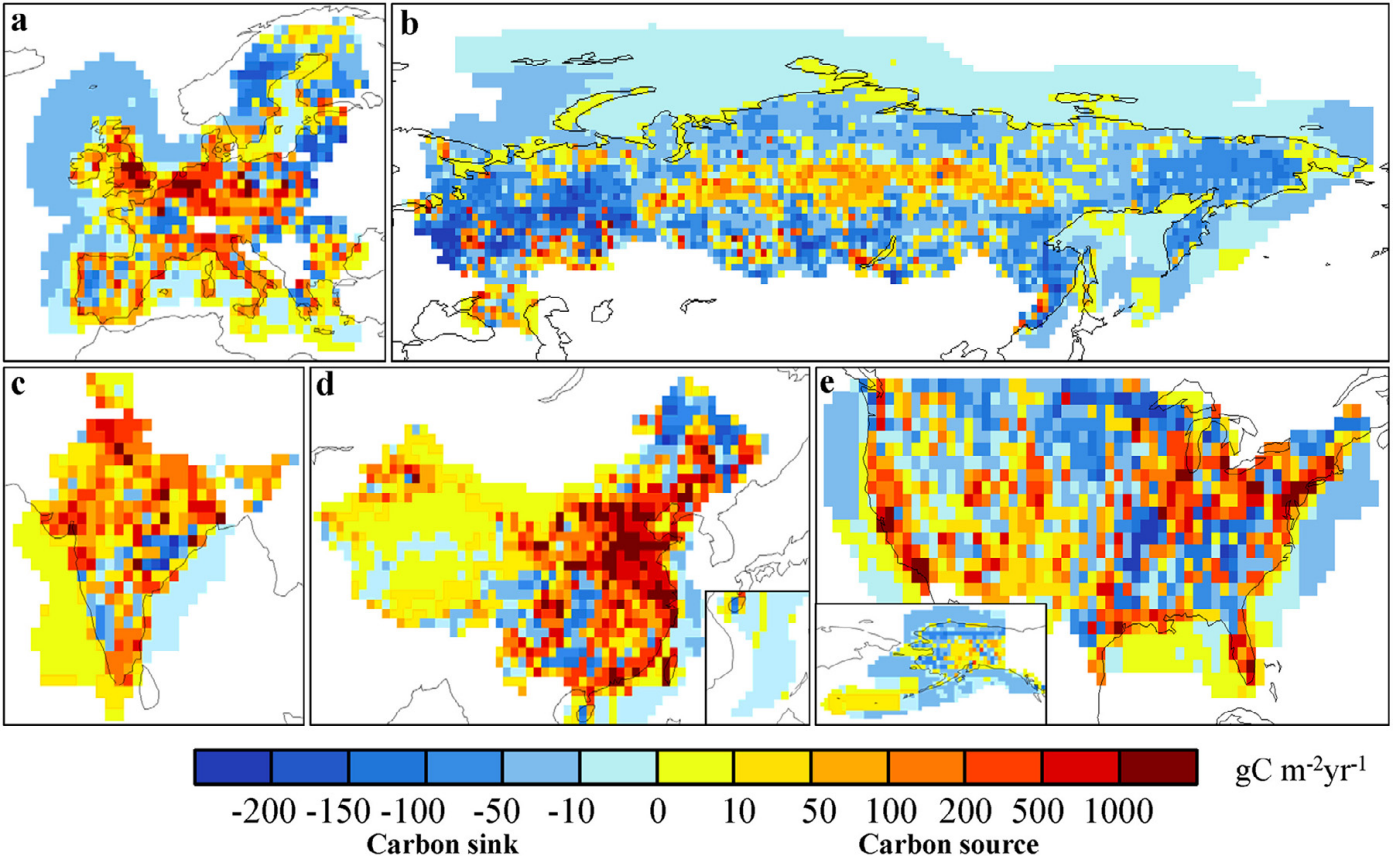
**Distributions of the posterior NCFs in the 5 CRs**.

**Fig. 7:**
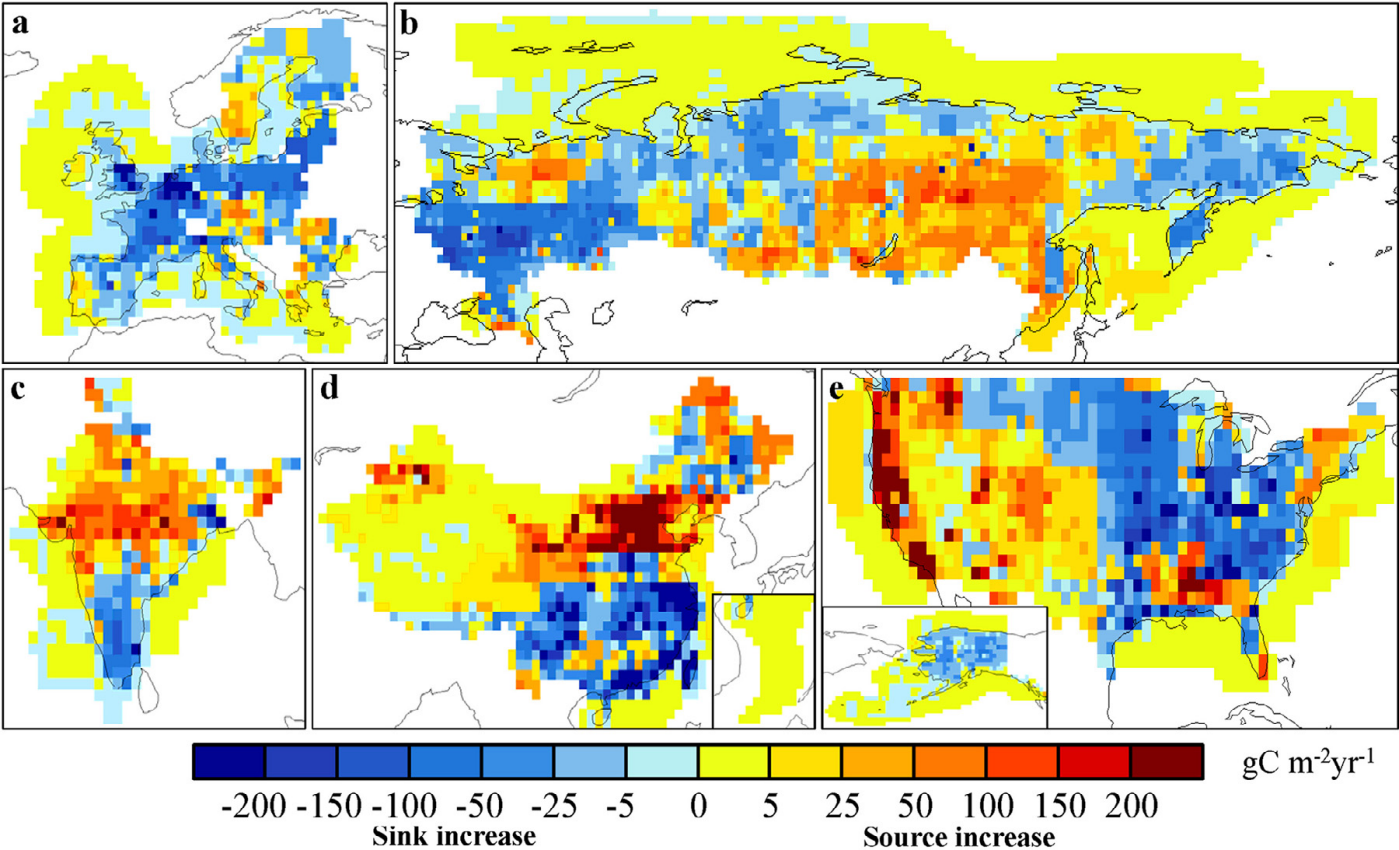
**Differences between the posterior and prior NCFs in the 5 CRs (Posterior minus prior)**.

In Fig. 6, we show the distributions of the mean posterior annual NCFs during 2010–2019 in the 5 CRs. In China, except for parts of the Northeast and Southwest, all other regions act as carbon sources, especially the North China Plain (NCP) and the Yangtze River Delta. In the US, parts of the central, northwest, and southeastern regions appear as carbon sinks, while obvious carbon sources are found in the northeast, California, and Florida. In the EU, the countries in northern Europe are represented as carbon sinks, while other countries in Europe are carbon sources, especially in the western Europe countries such as Germany, the Netherlands, and the United Kingdom. India is basically a carbon source in the whole country, only a few regions in the south are carbon sinks. In Russia, most regions of the country are carbon sinks, of which the western region is the strongest. Carbon sources are mainly distributed in central Siberia and urban areas. For the coastal ocean flux, Bohai and Taiwan Strait of China, most part of Gulf of Mexico of the US, Arabian Sea of India, and part of Mediterranean appear as weak carbon sources, while the other waters are weak carbon sinks. Compared with the prior NCF, after constrained with XCO_2_ retrievals, the carbon sinks are increased in southern China, the central and eastern US, most of the EU, southern India, and western Russia. In the meantime, the sources are increased in northern China, the western US, northern India, and most of Siberia, especially in the NCP of China and the West Coast (WC) of the US. Since there are weak NEE in NCP and WC, the significant increases of carbon sources in these areas indicate that the FCC emissions in these areas were underestimated (Fig. 7). Basu et al. [55] estimated the US's NCF in 2010 using surface CO_2_ and ^14^C observations, also showing that in the western US, the NCF was significantly underestimated, while in the eastern US, it was overestimated in the prior flux.

### 3.3 Interannual variations and trends of the NCFs in the 5 CRs

**Fig. 8:**
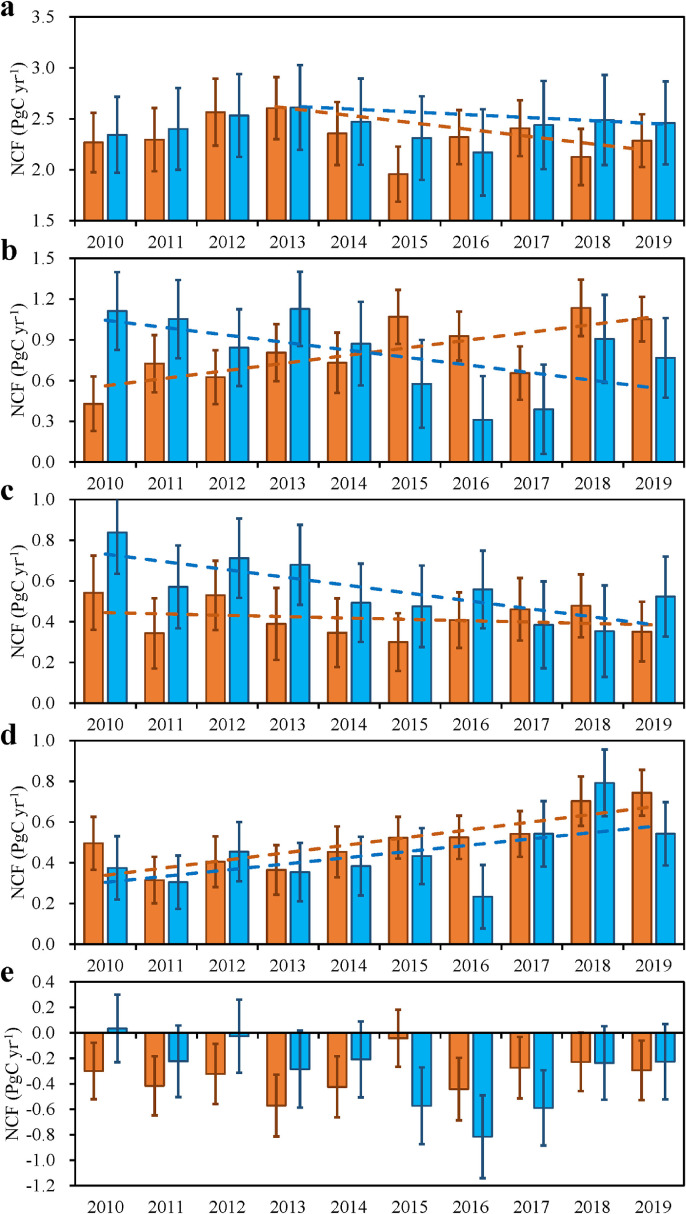
**Interannual variations of the posterior and prior NCFs in (a) China, (b) US, (c) EU, (d) India, and (e) Russia**.

From 2010 to 2019, the NCFs of the 5 CRs have significant interannual variations (Fig. 8). Except for Russia, the NCFs of the other 4 CRs are positive every year, indicating that they are all carbon sources. In China, from 2010 to 2013, the prior NCF increases year by year, and the posterior NCF increases at a larger rate. After 2013, the prior NCF shows a trend of initial decline and then increase, with the lowest in 2016, while the posterior NCF shows a slight downward trend with much fluctuation. In the US, the prior NCF shows an overall downward trend, with the lowest in 2016 and 2017, and a significant increase in 2018 and 2019, while the posterior NCF shows an overall upward trend from 2010 to 2019. In the EU, the prior NCF also shows a downward trend, but the posterior NCF does not show a clear trend and is relatively stable, basically around 0.5 PgC yr^−1^ every year. In India, the prior NCF is basically stable from 2010 to 2016, but it has increased significantly after 2016, while the posterior NCF shows an increasing trend year by year since 2013. In Russia, from 2010 to 2014, the prior NCF is a weak carbon source or a weak carbon sink. It is a strong carbon sink from 2015 to 2017, and turns into a weak carbon sink again in 2018 and 2019. The inter-annual changes of the posterior NCF are relatively small. Except for 2015 (almost neutral), the NCFs in Russia are all weak carbon sinks (-0.2 – -0.5 PgC yr^−1^). These indicate that the net contributions of the US and India to the global carbon budget have increased in recent 10 years, while in the EU, its contribution has been stable, while in China it has declined slightly after 2013.

The interannual variabilities are mainly dominated by the changes of NBE. Many studies have shown that NBE has large interannual variations, which are mainly caused by large-scale climate anomalies such as ENSO, and regional extreme climates such as drought and heatwave [21,56]. The relatively low NCFs in 2015 and 2018 in China may be related to the El Niño events that happened in these two years, especially in 2015. Previous study [57] showed that in the winter of the year when El Niño occurs, China tends to experience a warm winter; and in summer, the area south of the Yellow River will experience more precipitations, which are beneficial for the growth of vegetations, leading to a stronger carbon sink in these years. In the US, the year of 2017 was the third warmest year since record keeping began in 1895, the 20th wettest year on record, and had the smallest drought footprint in the past 18-years [58], resulting in a strong terrestrial carbon sink and relatively low NCF in this year. In the EU, the relatively high NCFs in 2010, 2012 and 2018 may be related to the widespread droughts happening in these years [59], [60], [61].

After 2013, China's FFC emissions are relatively stable [2], and the decline in its NCF should be caused by the increase in ecosystem carbon sink, which is supported by forest inventory data that showed an increase in the forest sink in China of 60% between 2009–2013 and 2014–2018 owing to intensive national afforestation / reforestation program [62]. In the US, the FFC emission had a slightly decreasing trend during the study period [2], thus the source increase in NCF should be attributed to the decline in ecosystem carbon uptakes. CMS-Flux also shows an upward trend of NCF from 2010 to 2018 in the US, with its value increased from 1.04 PgC yr^−1^ in 2010 to 1.19 PgC yr^−1^ in 2018, but CT2019B presents no clear trend. Being the largest contributor to the US land carbon sink, the forest carbon sequestration declined by 35% over 1973 – 2010, primarily because of stand ageing and a decline in forest area due to land use change [63]. Based on the US Environmental Protection Agency (EPA)’s National Greenhouse Gas inventory [64], the changes in forest carbon stocks declined about 5% from 2010 to 2019, which partly confirms the decline in the ecosystem carbon uptake during the study period. The increase of NCF in India (by 51%) is mainly due to the increase in the FFC emission by 56% from 2010 to 2019 [2].

**Fig. 9:**
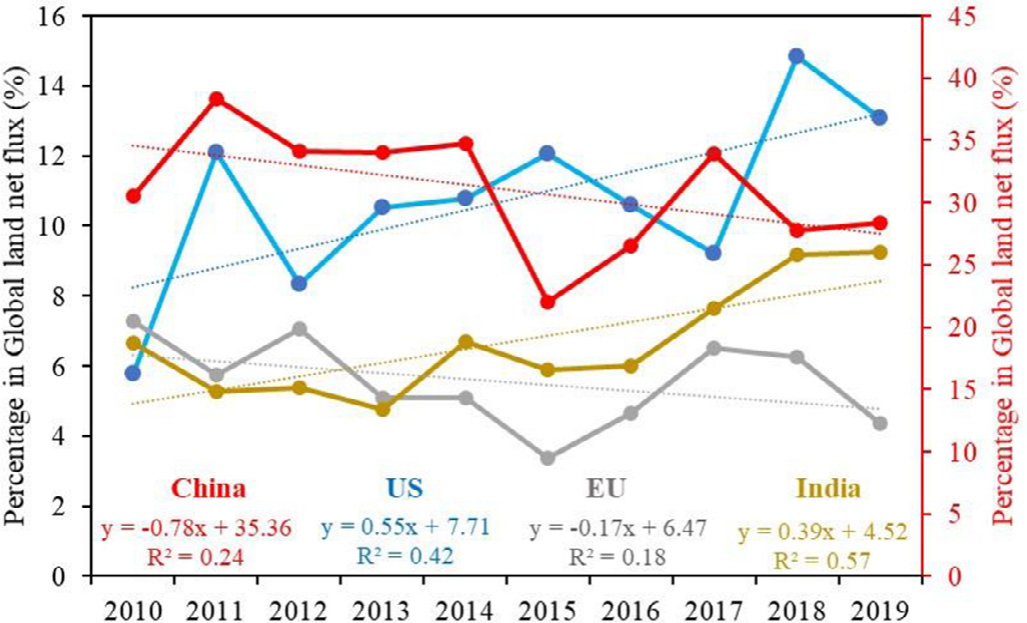
**Interannual variations of the contributions of each CR's NCF to the global land NCF, the dotted line plots the linear trend over the time series**.

We also calculated the percentage of each CR's posterior NCF in the global land NCF every year (Fig. 9), which reflects the relative contribution of each CR to the global carbon budget. Since Russia has already achieved carbon neutrality (Fig. 7), its relative contribution is not shown here. The global land NCF was obtained from GCP2020, which is the sum of AGR and ocean sink. On average, the percentage of China, the US, the EU, and India is 31.0%, 10.7%, 5.5% and 6.7%, respectively. From 2010 to 2019, China and the EU have downward trends, with slopes of -0.78 and -0.17 percent per year, respectively. Their contributions decrease from about 34 and 6.7% in the first 3 years to about 30 and 5.7% in the last 3 years. On the contrary, the US and India have increasing trends, with slopes of 0.55 and 0.39 percent per year, and their contributions increase from about 9% and 5.8% to 12% and 8.7%, respectively. This shows that the relative contribution of China and the EU to the global atmospheric CO_2_ concentration has been declining in the past 10 years, whereas the US and India have shown an upward trend, suggesting that the US and India should take more active measures to control anthropogenic CO_2_ emissions or increase carbon sinks.

## 4 Summary and conclusion

In this study, we used both GOSAT and OCO-2 XCO_2_ retrievals to constrain global surface NCFs from May 2009 to December 2019 using the GCAS version 2. The system performance in the 5 CRs was shown through flux uncertainty reduction and XCO_2_ bias reduction, and the reliability of the posterior NCF was evaluated by comparing the posterior CO_2_ mixing ratios against observations from 11 surface flask sites in the 5 CRs. The annual URRs in China, the US, the EU, India, and Russia are comparable, with values of 29.9%, 34.2%, 20.2%, 21.3%, and 25.5%, respectively. Compared with the GOSAT XCO_2_ retrievals, the decreases of RMSE for the posterior XCO_2_ in the 5 CRs are also comparable, and are in the range of 61–70%. Independent evaluations using global AGRs and surface flask CO_2_ concentrations in China, the US, the EU, and Russia show that the posterior carbon fluxes could significantly improve the modeling of atmospheric CO_2_ concentrations at global scale and in these 4 CRs.

The mean annual posterior NCFs and their trends from 2010 to 2019 in the top 5 FFC emitting CRs were analyzed and discussed. We estimated that the mean annual NCFs in China, the US, the EU, India, and Russia from 2010 to 2019 are 2.33 ± 0.29, 0.82 ± 0.20, 0.42 ± 0.16, 0.50 ± 0.12, and -0.33 ± 0.23 PgC yr^−1^, respectively, indicating that Russia has achieved carbon neutrality, but the other 4 CRs are still far from being neutral. From 2010 to 2019, the NCFs of the US and India had increasing trends, while in the EU, that was stable, and in China, it showed a slight downward trend after 2013. The declining trend in China is mainly caused by the increase of NBE supported by forest inventory data, and the increasing trend in the US may be attributed to the decline in ecosystem carbon uptakes caused by the ageing of forest. The increase of NCF in India is consistent with the increasing rate of its FFC emission. These indicate satellite XCO_2_ retrievals could be used to monitor the changes of NCF in different regions around the world, which is of great significance for major countries to cope with climate change and achieve greenhouse gas control goals.

## Declaration of Competing Interest

The authors declare that they have no conflicts of interest in this work.
